# Complications of Diabetes and Metrics of Glycemic Management Derived From Continuous Glucose Monitoring

**DOI:** 10.1210/clinem/dgac034

**Published:** 2022-01-30

**Authors:** Michael Yapanis, Steven James, Maria E Craig, David O’Neal, Elif I Ekinci

**Affiliations:** 1 Department of Medicine, the University of Melbourne, Parkville 3052, Victoria, Australia; 2 Department of Endocrinology, Austin Health, Heidelberg 3084, Victoria, Australia; 3 School of Nursing, Midwifery and Paramedicine, the University of the Sunshine Coast, Petrie 4052, Queensland, Australia; 4 School of Clinical Medicine, UNSW Medicine and Health, Discipline of Paediatrics and Child Health, UNSW 2052, NSW, Australia; 5 The University of Sydney Children’s Hospital Westmead Clinical School, Westmead 2145, NSW, Australia; 6 Department of Endocrinology, St Vincent’s Hospital, Fitzroy 3065, Victoria, Australia

**Keywords:** continuous glucose monitoring, diabetes complications, glycemic variability, time-in-range, type 1 diabetes mellitus, type 2 diabetes mellitus

## Abstract

**Context:**

Although glycated hemoglobin A_1c_ is currently the best parameter used clinically to assess risk for the development of diabetes complications, it does not provide insight into short-term fluctuations in glucose levels. This review summarizes the relationship between continuous glucose monitoring (CGM)-derived metrics of glycemic variability and diabetes-related complications.

**Evidence Acquisition:**

PubMed and Embase databases were searched from January 1, 2010 to August 22, 2020, using the terms *type 1 diabetes*, *type 2 diabetes*, *diabetes-related microvascular and macrovascular complications*, and *measures of glycaemic variability*. Exclusion criteria were studies that did not use CGM and studies involving participants who were not diabetic, acutely unwell (post stroke, post surgery), pregnant, or using insulin pumps.

**Evidence Synthesis:**

A total of 1636 records were identified, and 1602 were excluded, leaving 34 publications in the final review. Of the 20 852 total participants, 663 had type 1 diabetes (T1D) and 19 909 had type 2 diabetes (T2D). Glycemic variability and low time in range (TIR) showed associations with all studied microvascular and macrovascular complications of diabetes. Notably, higher TIR was associated with reduced risk of albuminuria, retinopathy, cardiovascular disease mortality, all-cause mortality, and abnormal carotid intima-media thickness. Peripheral neuropathy was predominantly associated with standard deviation of blood glucose levels (SD) and mean amplitude of glycemic excursions (MAGE).

**Conclusion:**

The evidence supports the association between diabetes complications and CGM-derived measures of intraday glycemic variability. TIR emerged as the most consistent measure, supporting its emerging role in clinical practice. More longitudinal studies and trials are required to confirm these associations, particularly for T1D, for which there are limited data.

Diabetes is associated with microvascular and macrovascular complications, including nephropathy, retinopathy, neuropathy, and cardiovascular and cerebrovascular disease, all of which contribute to a burgeoning disease burden. The risk of cardiovascular disease mortality and incidence of stroke are 2 to 4 times higher in Americans with diabetes compared to those without diabetes (1). Diabetic nephropathy accounts for 38.6% of new cases of end-stage renal disease, and 11.7% of adults with diabetes reported vision disability due to diabetic retinopathy (2). Globally, up to 75% of all lower-extremity amputations are performed in individuals with diabetes (3). In 2019, diabetes-related costs were estimated to have totaled $760 billion globally and $161.4 billion in Europe, with these figures being primarily composed of preventable hospital-based admissions from diabetes complications (4). Beyond financial cost, diabetes has a major effect on quality of life because of the daily demands of disease self-management, and possible effect of living with diabetes-related complications (5).

Since the release of findings from the landmark Diabetes Control and Complications Trial (DCCT) and United Kingdom Prospective Diabetes Studies (UKPDS), glycated hemoglobin A_1c_ (HbA_1c_) has become the gold standard by which to assess the success of glycemic management, and is used to guide clinical decision-making in diabetes management (6). There is a substantial body of evidence linking increases in HbA_1c_ with diabetes-related complications. Yet patients with identical HbA_1c_ values can also have vastly different complications rates—for example, only 11% of the variation in retinopathy risk may be explained by total glycemic exposure (HbA_1c_ and duration of diabetes) in the DCCT cohort (7, 8). Another study found mean HbA_1c_ to be only weakly correlated to the presence and severity of cardiovascular autonomic neuropathy (*r* = 0.22) (9). In addition, HbA_1c_ measurements may also be influenced by a number of common pathological and physiological factors unrelated to glucose levels such as age, race, iron-deficiency anemia, chronic renal failure, pregnancy, and medications (10). While HbA_1c_ is an integral marker of glycemic exposure over the preceding 8 to 12 weeks, it cannot describe interday or intraday glucose fluctuations. Beck et al (11) showed there can be a wide range of glucose profiles associated with any given HbA_1c_ level. Limitations around HbA_1c_ highlight the need for complementary methods to assess glucose levels in people living with diabetes (Fig. 1).

**Figure 1.**
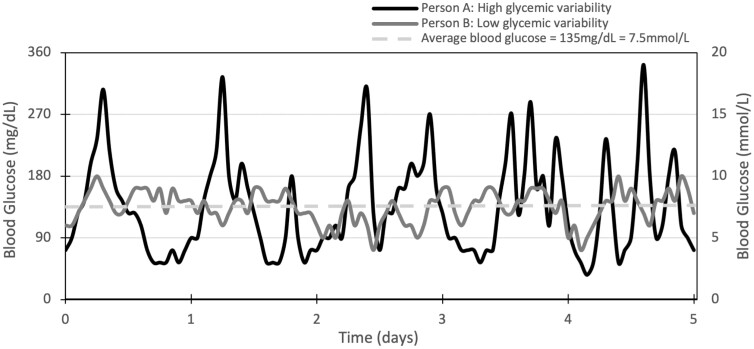
High vs low glycemic variability. Glucose profiles of 2 individuals showing identical glycated hemoglobin A_1c_ (6.3%) over a 5-day monitoring period but vastly different variability.

Continuous glucose monitoring (CGM) devices test interstitial glucose levels every 5 minutes and can record and store these data. Since the initial introduction of CGM into clinical practice in 2000 (12), the accuracy and sophistication of these devices has progressively increased. Over recent years, the use of CGM has become more widespread. In the United States their use in people with type 1 diabetes (T1D) has increased from approximately 7% in 2011 to 28% in 2017, according to the Type 1 Diabetes Exchange Clinic Network encompassing 81 diabetes centers (13). In Australia, CGM is used by 79% of people younger than 21 years. In 2017 the Advanced Technologies and Treatments for Diabetes (ATTD) Congress came to an international consensus that mean glucose, glucose management indicator (GMI), glycemic variability, TIR, time above range (TAR), and time below range (TBR) were the primary measurable outcomes of CGM; and that they should be measured for more than 14 days with at least 10 days of valid data (14). Glycemic variability itself can be measured by numerous formulas using CGM data, including SD, mean amplitude of glycemic excursions (MAGE), coefficient of variation for glucose (CV), high blood glucose index (HBGI), low blood glucose index (LBGI), area under the curve hypoglycemia, and continuous overall net glycemic action (CONGA). These metrics are summarized in Table 1. The congress recommends the use of CGM data to complement HbA_1c_ monitoring in a wide range of people with diabetes (14).

**Table 1.** Continuous glucose monitoring metrics

CGM metric	Description
TIR	Proportion of time spent with blood glucose levels within 3.9 to 10 mM. For most patients, a TIR of > 70% is an accepted target
TBR	Proportion of time spent with blood glucose levels below this range, with recommendations for < 4% of time spent with blood glucose levels 3.8 to 3.0 mM (level 1 TBR), and < 1% of time with blood glucose levels < 3.0 mM (level 2 TBR)
TAR	Proportion of time spent with blood glucose levels above this range, with recommendations for < 25% of time with blood glucose levels 10.1 to 13.9 mM (level 1 TAR), and < 5% of time > 13.9 mM (level 2 TAR)
SD	Measure of variation of all glucose measurements
MAGE	Measure of magnitudes of glycemic excursions (high and low) that exceed 1 SD from mean
CV	CV = (SD)/(mean glucose) × 100. CV < 36 is recommended (15)
CONGA	Combined measurement of timing and magnitude of blood glucose level fluctuations at specified time periods
GMI	Estimate of HbA_1c_ based on average glucose. Formerly known as estimated A_1c_

For more detail, see (16, 17).

Abbreviations: CGM, continuous glucose monitoring; CONGA, continuous overall net glycemic action; CV, coefficient of variation for glucose; GMI, glucose management indicator; HbA_1c_, glycated hemoglobin A_1c_; MAGE, mean amplitude of glycemic excursions, SD, SD of blood glucose levels; TAR, time above range; TBR, time below range; TIR, time in range.

CGM-derived outcomes are strongly correlated to HbA_1c_ and thus indirectly with diabetes-related complications by inference. Beck et al (18) found an *r* = –0.73 correlation between HbA_1c_ and TIR^70-180^. Hirsch et al (19) and Vigersky et al (20) correlated HbA_1c_ with TIR at *r* = –0.75 and *r* = –0.84, respectively. However, given the recency of the advent of CGM as a clinical and research tool, there is less evidence directly linking CGM metrics to these complications in comparison with HbA_1c_. This literature review therefore aims to amalgamate, summarize, and assess the existing evidence directly linking CGM-derived metrics with diabetes-related complications.

## Materials and Methods

A systematic literature search of PubMed and Embase was performed August 22, 2020, to identify studies demonstrating direct links between CGM-derived metrics of glycemic management, and diabetes-related complications. The search strategy is detailed in Table 2.

**Table 2.** Search strategy

Criteria	Terms included
1	*“Complications”* OR *“Microvascular”* OR *“Macrovascular”* OR *“Nephropathy”* OR *“Diabetic Kidney Disease”* OR *“End-Stage Kidney Disease”* OR *“End-stage renal disease”* OR *“Chronic Kidney Disease”* OR *Neuropathy* OR *Retinopathy* OR *“Eye disease”* OR *“CVD”* OR *“Cardiovascular disease”* OR *“Stroke”* OR *“CAD”* OR *“Coronary Artery Disease”* OR *“Cardiovascular Autonomic Neuropathy”* OR *“CAN”*
2	*“Target Range”* OR *“Time In Range”* OR *“TIR”* OR *“Glucose Variability”* OR *“GV”* OR *“Time Below Range”* OR *“TBR”* OR *“Time Above Range”* OR *“TAR”*
3	*“Diabetes Mellitus”* OR *“T1DM”* OR *“T2DM”* OR *“Type 1 Diabetes”* OR *“Type 2 Diabetes”* OR *“Diabetes Mellitus, Type 1”* OR *“Diabetes Mellitus, Type 2”*
4	1 AND 2 AND 3
5	Filters: English AND from 2010 (inclusive) to present

### Inclusion Criteria

Only studies from January 1, 2010 (inclusive) were considered because of the recent rapid development and usage of CGM, and the ATTD consensus on standardized CGM-derived metrics since 2017 (14). Data applicable to T1D and type 2 diabetes (T2D) and Latent Autoimmune Diabetes in Adults (LADA) were included to comprehensively capture all microvascular and macrovascular diabetes-related complications. Furthermore, markers of diabetes complications (eg, carotid intima-media thickness [CIMT] as a proxy for the development of macrovascular disease), were also included for the same reason (21, 22). Included studies were in humans of all ages and that were printed in English. Study types included were those presenting primary data.

### Exclusion Criteria

This review is designed to evaluate CGM, by which most devices measure glucose levels every 5 minutes. Studies using less frequent measurement regimens, such as the 7-point glucose profiles used in the DCCT, were excluded, as recent studies have suggested these glucose profiles are too infrequent to portray actual glycemic variability (23, 24). Baghurst et al (25) demonstrated that markers of acute glycemic variability, such as SD and MAGE, became unreliable if measured more than 2 to 4 hours and 1 hour apart, respectively.

The metrics used to evaluate glycemic management were those proposed at the ATTD Congress (14): TIR, TAR, TBR, CV, and GMI. All markers of intraday glycemic variability were included, which is the domain that distinguishes CGM from other glucose-monitoring regimens. Consequently, metrics not reliant on CGM, such as visit-to-visit glucose variability or HbA_1c_ variability, were excluded. Acutely unwell populations, for example postsurgery or poststroke patients, were excluded because of potentially altered glycemic management through mechanisms such as altered cortisol release (26). Studies in pregnant populations were also excluded (27). Publications involving insulin pump use were excluded because their effects on glycemic management may have altered the development of diabetes complications above the effect of CGM alone.

### Data Extraction and Synthesis

**Figure 2.**
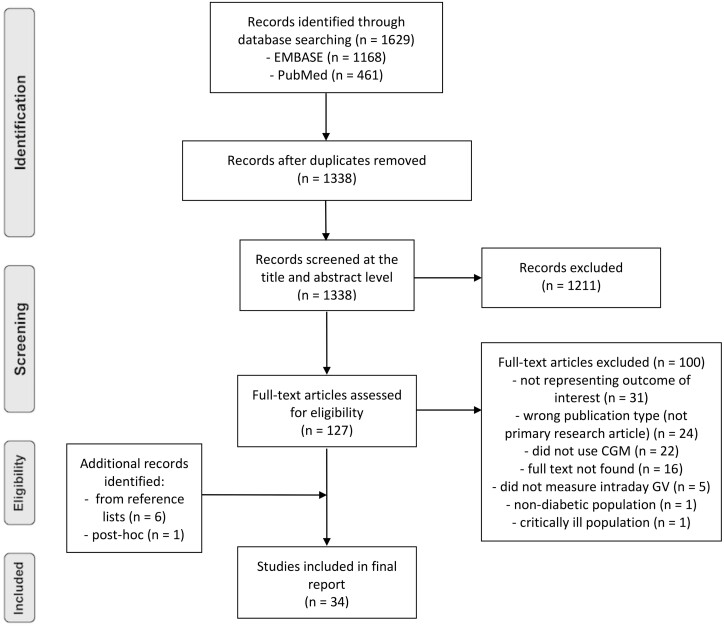
PRISMA (Preferred Reporting Items for Systematic Reviews and Meta-Analyses) flowchart.

A total of 1629 records were identified and exported to EndNote X9. Duplications were removed, and 1338 publications were screened by title and abstract (Fig. 2). The majority of papers excluded did not represent the outcome of interest. A total of 127 full-text papers were screened according to the defined criteria, and 27 met these criteria. A further 7 papers that met the criteria were identified: 6 via hand-searching reference lists, and 1 post hoc. The literature search identified conference abstracts that were subsequently hand-searched and included provided there was full-text availability. Data were then classified into T1D and T2D, and extracted into purpose-built tables.

Of the 34 included papers, 30 used cross-sectional study designs and 4 were longitudinal cohort studies. Nine studies involved participants with T1D, 22 with T2D, and 3 had mixed populations. Of the 20 852 total participants, 663 had T1D, 19 909 had T2D, 192 had LADA, and 88 were control participants without diabetes. Thirteen papers were from China, 9 from Europe, 4 from South Korea, 3 from Japan and the United States, and 2 from Australia. In addition, one multinational study included data from the United States, Europe, and Cameroon.

## Results

### Microvascular Complications

One study involving 32 participants addressed microvascular complications as a whole, showing associations with glycaemic variability (Table 3).

**Table 3.** Microvascular complications results

Study	Study size, No.	Population + age, y	Diabetes duration, y	Mean HbA_1c_, %	Duration of CGM trace, d	Findings
Šoupal et al (2014) (28) cross-sectional	32	T1D	19.5 ± 5.5	8.6 ± 0.9	12-14	Presence of any microvascular complication associated with
		41.5 ± 11.5				
						• SD: OR = 7.5 (1.83-52.08), *P* < .01^*b*^
						• MAGE: OR = 2.83 (1.3-8.17), *P* = .01^*b*^
						• CV: 0.43 ± 0.06 vs 0.38 ± 0.08, *P* = .03^*a*^

Values expressed as mean ± SD.

Abbreviations: CGM, continuous glucose monitoring; CV, coefficient of variation for glucose; HbA_1c_, glycated hemoglobin A_1c_; MAGE, mean amplitude of glycemic excursions; SD, SD of blood glucose levels; T1D, type 1 diabetes.

^*a*^Univariable analysis.

^*b*^Multivariable analysis.

#### Nephropathy

Six studies addressed nephropathy, involving 1563 participants (Table 4). Four of the 5 studies investigating albuminuria demonstrated statistically significant associations with glycemic variability. The larger studies (n = 866 and n = 281) found these associations with TIR. One study associated reduced eGFR (estimated glomerular filtration rate) with high SD.

**Table 4.** Nephropathy results

Study	Study size, No.	Population + age, y	Diabetes duration, y	Mean HbA_1c_, %	Duration of CGM trace, d	Findings
Šoupal et al (2014) (28) cross-sectional	32	T1D	19.5 ± 5.5	8.6 ± 0.9	12-14	Microalbuminuria associated with higher
		41.5 ± 11.5				• SD: 4.3 ± 0.5 vs 3.6 ± 0.8 mmol/L, *P* = .04^*a*^
						• CV: 0.46 ± 0.1 vs 0.39 ± 0.1 mmol/L, *P* = .02^*a*^
						• MAGE: 7.5 ± 0.9 vs 6.1 ± 1.2 mmol/L, *P* = .01^*a*^
Jin et al (2015) (29) cross-sectional	173	T2D	10.9 (6-16)	8.2 ± 3.7	3	Macroalbuminuria associated with higher
		56.7 ± 8.4				• SD: OR = 1.04 ± 0.04, *P* = .03^*b*^
						• MAGE: OR = 1.01 ± 0.01, *P* = .04^*a*^
Kuroda et al (2020) (30) longitudinal	281	T2D	13 (7-23)	6.9 (6.5-7.5)	10	Albumin-creatinine ratio associated with reduced TIR: β = –0.10, *P* = .04^*b*^
		68 (62-71)				
Magri et al (2018) (31) cross-sectional	121	T2D	3 (2-5)	6.8 (6.3-7.6)	3	Albuminuria not associated with TBR, TIR, or TAR
		64 (57-68)				
Yokota et al (2019) (32) cross-sectional	100	T2D	10 (0.1-42)	8.5 ± 1.9	3	Lower eGFR associated with high (≥ 35.9) SD: 66.2 ± 22.8 vs 78.8 ± 25.9, *P* = .01^*a*^
		60 ± 14				
Yoo et al (2020) (33) cross-sectional	866	T2D	13.1 ± 8.6	8.2 ± 1.5	3	Albuminuria risk associated with
		58.5 ± 10.3				• 10% lower TIR: OR = 0.94 (0.88-0.99), *P* = .04^*b*^
						• 10% higher TAR > 180 mg/dL: OR = 1.07 (1.01-1.19), *P* = .03^*b*^
						• 10% higher TAR > 250 mg/dL: OR = 1.10 (1.01-1.20), *P* = .03^*b*^

Values expressed as mean ± SD or median (interquartile range).

Abbreviations: CGM, continuous glucose monitoring; CV, coefficient of variation for glucose; eGFR, estimated glomerular filtration rate; HbA_1c_, glycated hemoglobin A_1c_; MAGE, mean amplitude of glycemic excursions; SD, SD of blood glucose levels; T1D, type 1 diabetes; T2D, type 2 diabetes; TAR, time above range; TBR, time below range; TIR, time in range.

^*a*^Univariable analysis.

^*b*^Multivariable analysis.

#### Retinopathy

Six studies addressed retinopathy, involving 6599 participants (Table 5). Four of the 5 studies investigating the presence of established retinopathy demonstrated statistically significant associations with glycemic variability. The larger studies (n = 3262 and n = 3119) found these associations for SD and reduced TIR. Both studies that addressed structural retinal changes in T1D found associations with glycemic variability, particularly LBGI.

**Table 5.** Retinopathy results

Study	Study size, No.	Population + age, y	Diabetes duration, y	Mean HbA_1c_, %	Duration of CGM trace, d	Findings
Picconi et al (2016) (34) cross-sectional	37	T1D	19.0 ± 10.4	7.9 ± 1.1	3	Inner nuclear layer thickness correlated with
		41.5 ± 10.0				• CONGA-1: *r* = 0.40, *P* = .03
						• CONGA-2: *r* = 0.39, *P* = .03
						• CONGA-4: *r* = 0.41, *P* = .02
						Retinal nerve fiber layer thickness correlated with LBGI: *r* = –0.38, *P* = .03
Sartore et al (2013) (35) cross-sectional	68	T1D, T2D	15.0 ± 8.3	8.1 ± 1.6	3	Retinopathy associated with
		48.6 ± 13.8				• SD: OR = 1.03 (1.01-1.06), *P* = .01^*a*^
						• CONGA-2: OR = 1.02(1.00-1.04), *P* = .04^*a*^
						• HBGI: OR = 1.10 (1.01-1.18), *P* = .03^*a*^
						Retinopathy not associated with MAGE: OR = 1.74 (0.69-4.40), *P* = .24^*a*^
Šoupal et al (2016) (28) cross-sectional	32	T1D	19.5 ± 5.5	8.6 ± 0.9	12-14	Retinopathy associated with SD: 4.1 ± 0.7 vs 3.5 ± 0.8 mmol/L, *P* = .03^*a*^
		41.5 ± 11.5				
Stem et al (2016) (36) cross-sectional	81	T1D	14.0 ± 6.7	7.9 ± 1.0	5	Neurodegenerative structural retinal changes were associated with
		46.5 ± 16.5				• LBGI: β = –0.47, *P* = .02, *R*^*2*^ = 0.28^*b*^
						• Area under curve for hypoglycemia: β = –0.45, *P* = .02, *R*^*2*^ = 0.26^*b*^
						Neither presence of retinopathy nor neuroretinal function associated with LBGI or area under curve for hypoglycemia
Lu et al (2018) (37) cross-sectional	3262	T2D	8.1 ± 6.8	8.9 ± 2.2	3	Retinopathy severity associated with
		60.2 ± 12.0				• Lower TIR: *P* < .01^*a*^
						• Lower TIR quartiles: *r* = –0.15, *P* < .01^*a*^
						• SD: *P* < .01^*a*^
						• CV: *P* < .01^*a*^
						• MAGE: *P* < 0.01^*a*^
						Any diabetic retinopathy negatively associated with 10% increase in TIR: OR = 0.92 (0.88-0.96), *P* < .01^*b*^
						Mild nonproliferative retinopathy negatively associated with
						• 10% increase in TIR: OR = 0.93 (0.87-0.99), *P* = .02^*b*^
						• Highest compared to lowest quartile TIR: OR = 0.56 (0.36-0.87), *P* = .01^*b*^
						Moderate nonproliferative retinopathy negatively associated with
						• 10% increase in TIR: OR = 0.91 (0.84-0.98), *P* = .01^*b*^
						• Highest compared to lowest quartile TIR: OR = 0.48 (0.27-0.83), *P* = .01^*b*^
						Vision-threatening retinopathy negatively associated with
						• 10% increase in TIR: OR = 0.91 (0.85-0.98), *P* = 0.02^*b*^
						• Highest compared to lowest quartile TIR: OR = 0.53 (0.30-0.91), *P* = .02^*b*^
Lu et al (2019) (38) cross-sectional	3119	T2D, LADA	7.7 ± 6.3	8.9 ± 2.1	3	Retinopathy associated with
		57.6 ± 10.1				• SD: OR = 1.15 (1.03-1.29), *P* = 0.02^*b*^
						• MAGE: OR = 1.21 (1.11-1.31), *P* < .01^*a*^
						• CV: OR = 1.16 (1.07-1.26), *P* < .01^*a*^
						Retinopathy associated with increasing quartiles of SD and MAGE in T2D: *P* < .01
						No significant associations for LADA

Values expressed as mean ± SD or median (interquartile range).

Abbreviations: CGM, continuous glucose monitoring; CONGA, continuous overall net glycemic action; HbA_1c_, glycated hemoglobin A_1c_; LADA, latent autoimmune diabetes of adulthood; LBGI, low blood glucose index; SD, SD of blood glucose levels; HBGI, high blood glucose index; MAGE, mean amplitude of glycemic excursions; T1D, type 1 diabetes; T2D, type 2 diabetes; TIR, time in range.

^*a*^Univariable analysis.

^*b*^Multivariable analysis.

#### Neuropathy

Peripheral neuropathy was investigated in 7 studies involving 2247 participants (Table 6). Four papers investigated the presence of peripheral neuropathy, while 3 studied markers of nerve conduction. Glycemic variability markers, particularly SD, MAGE, and reduced TIR, were associated both with the presence of peripheral neuropathy and abnormal nerve conduction across all papers. Cardiac autonomic neuropathy (CAN) was measured in 8 publications, involving 782 participants (Table 7). CAN was associated with glycemic variability in 7 of these publications, but some associations were directly contradicted in 4 publications. Reduced TIR was investigated in 2 studies and found to be associated with CAN in both.

**Table 6.** Peripheral neuropathy results

Study	Study size, No.	Population + age, y	Diabetes duration, y	Mean HbA_1c_, %	Duration of CGM trace, d	Findings
Kwai et al (2016) (39) cross-sectional	17	T1D	Not recorded	8.1 ± 0.3	6	Multiple measures of abnormal motor and sensory axonal function associated with MAGE
		28.6 ± 1.5				
						• Super excitability: *r* = 0.54, *P* = .04
						• S2 accommodation: *r* = –0.76, *P* < .01
						• Minimum current threshold (I/V) slope: *r* = 0.71, *P* < .01
						• Strength duration time constant: *r* = 0.66, *P* < .01
						• Latency: *r* = 0.65, *P* < .01
Šoupal et al (2014) (28) cross-sectional	32	T1D	19.5 ± 5.5	8.6 ± 0.9	12-14	Impaired vibration perception threshold associated with SD: *r* = 0.51, *P* < .01
		41.5 ± 11.5				
Kuroda et al (2020) (30) longitudinal	281	T2D	13 (7-23)	6.9 (6.5-7.5)	10	Peripheral neuropathy an explanatory factor for TIR: β = –0.11, *P* = .03^*b*^
		68 (62-71)				
Li et al (2020) (40) cross-sectional	740	T2D	10.7 ± 7.5	8.6 ± 1.9	3	Abnormal nerve conduction study markers negatively associated with highest TIR tertile
		60.2 ± 12.8				
						• Lower risk of slowing conduction velocity: OR = 0.26(0.18-0.40), *P* < .01^*b*^
						• Lower risk of amplitude reduction: OR = 0.60(0.41-0.88), *P* = .01^*b*^
						• Higher rate of reduced latency: OR = 1.71(1.16-2.53), *P* = .01^*b*^
Mayeda et al (2020) (41) cross-sectional	105	T2D	19.1 ± 10.0	7.8 ± 1.6	12	Michigan Neuropathy Screening Instrument questionnaire score ≥ 2 associated with 10% reduction in TIR: OR = 1.25 (1.02-1.52), *P* = .03^*b*^
		67.1 ± 10.0				
						Peripheral neuropathy associated with
						• TAR: OR = 1.24 (1.03-1.50), *P* = .02^*b*^
						• 1% increase in GMI: OR = 1.79 (1.05-3.04), *P* = .03^*b*^
						Peripheral neuropathy not associated with 6% increase in CV^*a*^
Hu et al (2018) (42) cross-sectional	982	T2D	5.2 (4.2-8.0)	9.9 ± 1.3	3	Peripheral neuropathy associated with
		55.1 ± 10.9				• SD: OR = 3.71 (2.61-5.28), *P* < .01^*a*^
						• MAGE: OR = 4.57 (3.48-6.10), *P* < .01^*b*^
Xu et al (2014) (43) cross-sectional	90	T2D	5.5 (2-8.5)	6.5 ± 0.4	3	Peripheral neuropathy associated with:
		59.3 ± 7.5				• SD: OR = 2.95 (1.55-5.61), *P* < .01^*a*^
						• MAGE: OR = 2.05 (1.36-3.09), *P* < .01^*b*^

Values expressed as mean ± SD or median (interquartile range).

Abbreviations: CGM, continuous glucose monitoring; CV, coefficient of variation for glucose; GMI, Glucose Management Index; HbA_1c_, glycated hemoglobin A_1c_; MAGE, mean amplitude of glycemic excursions; SD, SD of blood glucose levels; T1D, type 1 diabetes; T2D, type 2 diabetes; TAR, time above range; TIR, time in range;

^*a*^Univariable analysis.

^*b*^Multivariable analysis.

**Table 7.** Cardiac autonomic neuropathy results

Study	Study size, No.	Population + age, y	Diabetes duration, y	Mean HbA_1c_, %	Duration of CGM trace, d	Findings
Jun et al (2019) (44) cross-sectional	80	T1D	10.1 ± 7.3	8.2 ± 1.7	3	CAN associated with
		39.9 ± 14.0				• Reduced TIR: 40.0 (26.3-53.2) vs 57.0 (41.1-72.2), *P* < .01^*a*^
						• TBR: 5.1 (0.0-15.7) vs 1.7 (0.0-4.6), *P* = 0.01^*a*^
						• SD: OR = 1.05 (1.02-1.09), *P* = .01^*b*^
						• MAGE: OR = 1.02 (1.01-1.03), *P* = .02^*b*^
						• CV: OR = 1.11 (1.05-1.18), *P* < .01^*b*^
						• LBGI: OR = 1.29 (1.11-1.49), *P* < .01^*b*^
						• HBGI: OR = 1.23 (1.05-1.43), *P* = .01^*b*^
						• Log(TIR + 1): OR = 0.08 (0.01-0.58), *P* = .03^*b*^
						• Log(TBR + 1): OR = 15.1 (3.33-68.57), *P* < .01^*b*^
						• Log(TBR < 54 mg/dL + 1): OR = 38.6 (6.35-234.7), *P* < .01^*b*^
Nyiraty et al (2018) (45) cross-sectional	20	T1D	17.5 ± 2.5	8.1 ± 0.2	6	CAN severity associated with SD: *r* = 0.49, *P* < .05^*b*^
		39.5 ± 3.4				Presence of CAN was not associated with SD, MAGE or CONGA^*a*^
Di Flaviani et al (2010) (46) cross-sectional	26	T2D	4.4 ± 4.8	6.7 ± 1.3	1	Abnormal sympathovagal balance (increased LF/HF ratio) associated with MAGE only at nighttime: r = 0.40, *P* = .04^*a*^
		59.2 ± 10.6				
Guo et al (2020) (47) cross-sectional	349	T2D	6 (2-12)	9.2 ± 2.3	3	CAN severity associated with SD: *P* < .01^*a*^
		53.1 ± 12.9				Manifest CAN negatively associated with TIR: OR = 0.97 (0.95-0.98), *P* < .01^*b*^
						Severe CAN negatively associated with TIR: OR = 0.94 (0.91-0.98), *P* < .01^*b*^
Jun et al (2015) (48) cross-sectional	110	T2D	12.8 ± 7.1	7.9 ± 1.0	3	CAN associated with
		58.1 ± 8.4				• SD: OR = 1.04 (1.01-1.07), *P* < .01^*a*^
						• CV: OR = 1.07 (1.01-1.13), *P* = .03^*b*^
						No association with MAGE: OR = 1.01 (0.99-1.02), *P* = .06^*a*^
Kalopita et al (2014) (49) cross-sectional	50	T2D	5.5 (2.0-9.3)	7.1 ± 3.3	1	CAN, as measured by abnormal indices of heart rate variability on ECG, not associated with SD or MAGE
		58.4 ± 9.9				
Matsutani et al (2018) (50) longitudinal	57	T2D	11.5 ± 9.6	7.3 ± 1.0	3	Baroreflex sensitivity associated with
		67.2 ± 7.7				• CV: β = –0.31, *P* = .03^*b*^
						• SD: *r* = –0.37, *P* = .01^*a*^
Xu et al (2016) (51) cross-sectional	90	T2D	Not recorded	9.3 ± 2.1	3	CAN associated with MAGE: OR = 1.73 (1.01-2.73), *P* = .02^*b*^
		46.7 ± 10.0				CAN not associated with CV

Values expressed as mean ± SD or median (interquartile range).

Abbreviations: CAN, cardiac autonomic neuropathy; CGM, continuous glucose monitoring; CONGA, continuous overall net glycemic action; CV, coefficient of variation for glucose; ECG, electrocardiography; HbA_1c_, glycated hemoglobin A_1c_; HBGI, high blood glucose index; LBGI, low blood glucose index; MAGE, mean amplitude of glycemic excursions; SD, SD of blood glucose levels; T1D, type 1 diabetes; T2D, type 2 diabetes; TBR, time below range; TIR, time in range.

^*a*^Univariable analysis.

^*b*^Multivariable analysis.

### Macrovascular Disease

Thirteen studies addressed different aspects of macrovascular disease, which encompasses cardiovascular, cerebrovascular, and peripheral vascular disease, involving 10 206 participants: 412 with T1D and 9794 with T2D (Tables 8 and 9). Four papers investigated the presence of established macrovascular disease in which MAGE and reduced TIR were the predominant associations. The large (n = 6225) prospective cohort study identified TIR as an association for cardiovascular disease mortality and all-cause mortality. Glycemic variability was associated with abnormal echocardiography finding in two studies. Three studies used angiography to measure coronary artery disease in which glycemic variability, particularly MAGE, was an association. Cardiovascular disease risk factors were associated with glycemic variability in 1 out of 2 studies. Endothelial function was investigated in 1 paper and generally was not statistically significantly correlated with glycemic variability. CIMT was evaluated as a well-established proxy for cardiovascular and cerebrovascular disease (21, 22) in 4 publications. Associations with MAGE and SD were varied, but associations with TIR and TBR were unopposed.

**Table 8.** Macrovascular disease results

Study	Study size, No.	Population + age, y	Diabetes duration, y	Mean HbA_1c_, %	Duration of CGM trace, d	Findings
Borg et al (2011) (52) longitudinal	427	T1D, T2D	Not recorded	6.8 ± 1.3	> 2 d, 4 separate times	Cardiovascular disease risk factors (lipid profile, blood pressure, CRP) not associated with SD, MAGE, or CONGA
		46 ± 14				
Peña et al (2012) (53) cross-sectional	52	T1D	5.5 ± 4	8.9 (6.7-14)	2	Endothelial function, measured by low-mediated dilatation, inversely correlated with LBGI: *r* = –0.30, *P* = .03
		14 (2.7)				
						Not significantly associated with
						• SD: *r* = 0.16, *P* > .05^*a*^
						• MAGE: *r* = –0.06, *P* > .05^*a*^
						• CONGA-1: *r* = –0.04, *P* > .05^*a*^
						• CONGA-4: *r* = 0.04, *P* > .05^*a*^
						• CONGA-8: *r* = –0.05, *P* > .05^*a*^
Snell-Bergeon et al (2010) (54) cross-sectional	75	T1D	29 ± 8	7.4 ± 0.9	5	Coronary artery calcium associated with
		42 ± 9				• TAR: OR = 5.5 (1.3-22.6), *P* = .02^*b*^
						• Time-out-of-range: OR = 5.7 (1.3-24.9), *P* = .02^*b*^
						• SD in men only: OR = 4.7 (1.1-19.7), *P* = 0.03^*b*^
						Log coronary artery calcification score associated with
						• Time out of range: *r* =* *0.41, *P* = .03^*b*^
						• TAR: *r* =* *0.47, *P* = .01^*b*^
Di Flaviani et al (2010) (46) cross-sectional	26	T2D	4.4 ± 4.8	6.7 ± 1.3	1	Left ventricular mass index correlated with CONGA-2: *r* = 0.55, *P* = .01^*a*^
		59.2 ± 10.6				
Lu et al (2020) (55) longitudinal	6,225	T2D	9.7 ± 7.4	8.9 ± 2.2	3	Cardiovascular disease mortality associated with
		61.7 ± 11.9				• TIR 71%-85%: HR = 1.35 (0.90-2.04), *P* = .02^*b*^
						• TIR 51%-70%: HR = 1.47 (0.99-2.19), *P* = .02^*b*^
						• TIR < 50%: HR = 1.85 (1.25-2.72), *P* = .02^*b*^
						• 10% decrease in TIR: HR = 1.05 (1.00–1.11), *P* = .02^*b*^
						All-cause mortality associated with
						• TIR 71%-85%: HR = 1.23 (0.98-1.55), *P* < .01^*b*^
						• TIR 51%-70%: HR = 1.30 (1.04-1.63), *P* < .01^*b*^
						• TIR < 50%: HR = 1.83 (1.48-2.28), *P* < .01^*b*^
						• 10% decrease in TIR: HR = 1.08 (1.05-1.12), *P* < .01^*b*^
Magri et al (2018) (31) cross-sectional	121	T2D	3 (2-5)	6.8 (6.3-7.6)	3	Macrovascular disease associated with TBR: OR = 1.12 (1.01-1.23), *P* = .02^*b*^
		64 (57-68)				
						Macrovascular disease not associated with TIR: *P* = 0.63^*b*^ or TAR: *P* = .39^*b*^
Su et al (2011) (56) cross-sectional	344	T2D	6.1 ± 6.2	7.6 ± 1.5	3	Coronary artery disease associated with
		63.9 ± 9.0				• MAGE: 3.7 ± 1.4 vs 3.2 ± 1.2 mmol/L, *P* < .01^*a*^
						• MAGE ≥ 3.4 mmol/L: OR = 2.61 (1.41-4.83), *P* < .01^*b*^
						Gensini score (measure of coronary artery disease severity) correlated with MAGE: *R*^2^ = 0.19, *r* = 0.28, *P* < .01^*b*^
Tang et al (2016) (57) cross-sectional	240	T2D	5.7 ± 6.2	6.1 ± 0.9	3	Framingham risk score (10-y cardiovascular disease risk) correlated with
		51.9 ± 8.0				
						• SD: *r* = 0.51, *P* < .01
						• MAGE: *r* = 0.49, *P* < .01
						Framingham risk score > 20% (high 10-y cardiovascular disease risk) associated with
						• SD: OR = 1.22, *P* = .04^*a*^
						• MAGE: OR = 1.62 (1.20-2.32), *P* < .01^*b*^
Yokota et al (2019) (32) cross-sectional	100	T2D	10 (0.1-42)	8.5 ± 1.9	3	Reduced left ventricular diastolic function associated with high (≥ 35.9 mg/dL) SD: OR = 3.67 (1.02-13.22), *P* < .05^*b*^
		60 ± 14				
Zhang et al (2013) (58) cross-sectional	148	T2D 59.6 ± 7.0	Not recorded	7.2 ± 1.3	3	Cardiovascular complications associated with
						• MAGE: 4.0 (3.3-4.8) vs. 2.6 (1.9-3.5), *P* < .01^*a*^
						• SD: 2.0 ± 0.8 vs 0.1.5 ± 0.4, *P* < .05^*a*^
						SYNTAX scores (a complete angiography scoring system) statistically significantly correlated to MAGE: *r* = 0.52, *P* = .01^*b*^
						Coronary intima-media thickness correlated with MAGE: *r* = 0.46, *P* < .01

Values expressed as mean ± SD or median (interquartile range).

Abbreviations: CGM, continuous glucose monitoring; CONGA, continuous overall net glycemic action; CRP, C-reactive protein; HBGI, high blood glucose index; LBGI, low blood glucose index; MAGE, mean amplitude of glycemic excursions; SD, SD of blood glucose levels; T1D, type 1 diabetes; T2D, type 2 diabetes; TAR, time above range; TBR, time below range; TIR, time in range.

^*a*^Univariable analysis.

^*b*^Multivariable analysis.

**Table 9.** Carotid intima-media thickness results

Study	Study size, No.	Population + age, y	Diabetes duration, y	Mean HbA_1c_, %	Duration of CGM trace, d	Findings
Cesana et al (2013) (59) cross-sectional	17	T1D	13.6 ± 8.8	7.7 ± 1.2	1	CIMT not correlated with SD or MAGE
		40.7 ± 7.5				
Lu et al (2020) (60) cross-sectional	2,215	T2D	8.5 ± 6.7	8.9 ± 2.1	3	Abnormal (≥ 1 mm) CIMT associated with
		60.4 ± 11.5				• SD: 2.3 ± 0.9 vs 2.5 ± 0.9, *P* = .01^*a*^
						• MAGE: 5.8 ± 2.5 vs 6.3 ± 2.7, *P* = .01^*a*^
						• Lower TIR: 66.4 ± 23.5 vs 60.7 ± 24.9, *P* < .01^*a*^
						Abnormal CIMT not associated with CV: *P* = .33^*a*^
						Abnormal CIMT negatively associated with 10% higher TIR: OR = 0.94 (0.88-1.00), *P* = .04^*b*^
Magri et al (2018) (31) cross-sectional	121	T2D	3 (2-5)	6.8 (6.3-7.6)	3	Abnormal CIMT associated with TBR: OR = 1.09 (1.00-1.19), *P* = .04^*b*^
		64 (57-68)				
Mo et al (2013) (61) cross-sectional	216	T2D	9 (5-13.3)	8.3 ± 1.7	3	Intracranial/cervical artery stenosis severity, measured by magnetic resonance angiography, not associated with SD or MAGE
		63 ± 10				
						In participants without existing plaques found on magnetic resonance angiography, CIMT correlated with
						• SD: standardized β = 0.34, *P* = .01, *R*^2^ = 0.31^*b*^
						• MAGE: standardized β = 0.32, *P* = .01, *R*^2^ = 0.27^*b*^
						In those with existing atherosclerotic plaque, CIMT not correlated with SD or MAGE

Values expressed as mean ± SD or median (interquartile range).

Abbreviations: CGM, continuous glucose monitoring; CIMT, carotid intima-media thickness; CV, coefficient of variation for glucose; HbA_1c_, glycated hemoglobin A_1c_; MAGE, mean amplitude of glycemic excursions; SD, SD of blood glucose levels; T1D, type 1 diabetes; T2D, type 2 diabetes; TBR, time below range; TIR, time in range;.

^*a*^Univariable analysis.

^*b*^Multivariable analysis.

### Glycemic Variability Metrics and Glycated Hemoglobin A_1c_

Overall, 22 out of 34 studies investigated the associations between CGM metrics and diabetes complications after adjusting for HbA_1c_ (Table 10). Most (n = 19) showed various CGM metrics (namely MAGE, TIR, CV, and TBR) remained associated with diabetes complications after adjusting for HbA_1c_. Five studies found a glycemic variability metric to lose significance after adjustment for HbA_1c_.

**Table 10.** Glycemic variability metrics and glycated hemoglobin A_1c_

CGM marker	No. of papers showing associations of glucose metrics with diabetes complications after adjusting for HbA_1c_	No. of papers in which statistical significance was lost after adjusting for HbA_1c_
SD	7	3
MAGE	7	1
TIR (and time-out-of-range)	5	1
CV	3	0
TBR (and AUC TBR)	2	0
LBGI	2	0
CONGA-2	0	2
HBGI	1	1
TAR	1	0

Abbreviations: AUC, area under the curve; CGM, continuous glucose monitoring; CONGA, continuous overall net glycemic action; CV, coefficient of variation for glucose; HbA_1c_, glycated hemoglobin A_1c_; HBGI, high blood glucose index; LGBI, low blood glucose index; MAGE, mean amplitude of glycemic excursions; SD, SD of blood glucose levels; TAR, time above range; TBR, time below range; TIR, time in range.

Complete findings from each individual study can be found in Tables S4 (62).

## Discussion

This review of 34 publications totaling 20 852 participants, which investigated the associations between 6 different domains of diabetes complications against more than 10 different markers of intraday glycemic variability, demonstrated associations with all included diabetes complications. Glycemic variability, particularly low TIR and high SD, was almost unanimously associated with nephropathy, retinopathy, and peripheral neuropathy. Associations with CAN were present but varied. Glycemic variability was also associated with the presence of cardiovascular disease, cardiovascular disease mortality, all-cause mortality, and CIMT, as well as echocardiography and angiography abnormalities.

Of the CGM metrics recommended at the ATTD Congress, TIR was identified as the single most important CGM metric as it provides the most clinically practical information in 11 studies involving 14 319 participants (14). In nephropathy, TIR and glycemic variability metrics were strongly supported except for one cross-sectional study. The evidence for TIR and glycemic variability metrics in retinopathy was particularly strong given the study sizes (n = 6381) (37, 38). Glycemic variability was also associated with all severities of retinopathy, including preclinical neuroretinal abnormalities. Benbow et al (63) found peripheral neuropathy to be the most common complication of diabetes, having a major effect on quality of life, and CAN is a particularly strong risk factor for mortality in T1D (64, 65). Overall, the evidence suggests CAN is associated with decreased TIR, but not glycemic variability; but in peripheral neuropathy both glycemic variability—particularly MAGE—and lower TIR were supported as risk factors. These results highlight the importance of measuring these intraday parameters.

Approximately three-quarters of individuals with diabetes die from a cardiovascular cause (66), and a diagnosis of diabetes may be as much a risk factor for poor cardiovascular disease outcomes as having coronary artery disease itself (67), even in T1D (68). Most evidence supported lower TIR as a risk factor for macrovascular disease. The largest study was of longitudinal study design and importantly provided strong evidence for TIR as a protective factor for cardiovascular disease mortality (55). The second largest was a cross-sectional study that also showed strong correlations between TIR and CIMT. Only one cross-sectional study that measured the presence of overt macrovascular disease found no statistically significant association with TIR.

**Figure 3.**
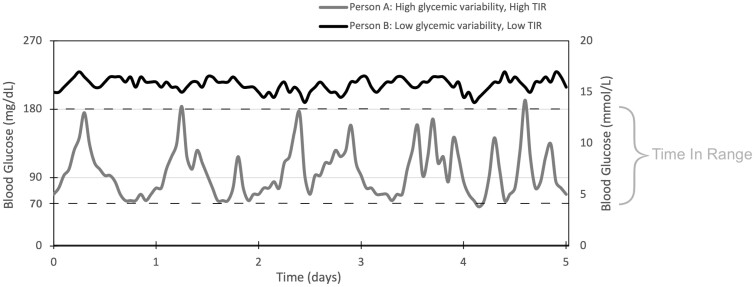
Time in range (TIR) vs glycemic variability. Glucose profiles of 2 individuals highlighting the difference between glycemic variability and TIR.

Glycemic variability showed mixed results for macrovascular disease and tended to relate more to microvascular complications. The ATTD Congress decided on CV as the consensus marker of glycemic variability (14), as SD is flawed in that it is significantly influenced by mean glucose. Yet CV seemed to have the least statistically significant associations with complications of diabetes, although it was measured only in 12 studies. While TIR and CV are emerging as popular CGM metrics, the relationship between the two should also be elucidated as it is possible to have a highly variable CGM trace with a high TIR, and conversely a minimally variable trace with low TIR (Fig. 3). Three studies investigated this in T2D, finding TIR remaining associated with albuminuria, retinopathy, and CAN after adjustments for glycemic variability metrics such as SD, MAGE, and CV (33, 37, 47). No studies examined the association of glycemic variability metrics after adjustment for TIR. Further research is required to clarify the nature of the relationship between TIR and CV with overt diabetes-related complications. Ideally these would involve large-scale longitudinal studies or randomized controlled trials (RCTs) with consistent CGM data that are long enough to capture adequate numbers of outcome events. However, such studies would be expensive and difficult to standardize for confounders such as exercise, physical activity, and treatment. Rather than individually randomized trials, evidence from CGM data using artificial intelligence may assist in interpreting observational data from individuals using CGM in collaboration with producers of CGM.

Another reason for the lack of definitive outcomes is this review’s strict inclusion criteria of CGM use, as opposed to other less frequent blood glucose level (BGL) measurement regimens. While the accuracy of these infrequent measurement regimens is questionable compared to CGM (23-25), there is a wealth of data that must be considered. The DCCT was a large multicenter RCT spanning 10 years and involving 1440 participants. Reanalysis from Beck et al (69, 70) found that every 10% increase in TIR reduced the risk of retinopathy and microalbuminuria by 64% and 40%, respectively. However, the DCCT commenced in 1982 when therapeutic regimens and technologies were very different compared with today. It did not use CGM, and BGLs were measured only via 7 finger-prick samples collected in 1 day, every 3 months.

### Mechanisms Relating Glycemic Variability With Diabetes Complications

Since diabetes complication risk is only partly explained by HbA_1c_ (8), intraday glycemic variability is important to measure in conjunction with average glycemia because of its independent pathogeneses summarized by Nusca et al (16). While chronic sustained hyperglycemia can cause excessive protein glycation, acute hyperglycemic fluctuations may cause increased oxidative stress, inflammation, endothelial dysfunction, and altered gene expression (71-75). One case-control study involving 27 participants with T2D investigated the effect of glycemic variability on endothelial function (measured by flow-mediated dilatation) and oxidative stress (measured by plasma 3-nitrotyrosine and 8-iso-PGF2α urinary excretion rates). They found that oscillations in BGLs resulted in statistically significantly more endothelial dysfunction and oxidative stress than constant glucose levels, even if the average glucose was higher in the stable group. These effects lasted even beyond the return to euglycemia; thus, highlighting the importance of glycemic variability (72). A case-control study confirmed the strong association (*r* = 0.86) between MAGE and 8-iso-PGF2α urinary excretion rates (75), while another showed that transient hyperglycemia can induce lasting epigenetic changes in the promotor region of *NFκB* (a proinflammatory gene) in vitro and in mice (73). It is also established that high glycemia variability is associated with more frequent episodes of hypoglycemia, which contributes to a range of adverse effects including cardiovascular morbidity and mortality (76).

A narrative review by Livingstone et al (76) explored the glucose variability hypothesis—the notion that glucose variability contributes additional risk of diabetes complications after adjusting for HbA_1c_. While they agreed mechanisms for glycemic variability causing complications exist, they concluded that there were insufficient data at the time to substantiate this hypothesis. However, this was based primarily on the DCCT/EDIC (Epidemiology of Diabetes Interventions and Complications), which was greatly limited in that it used 7-point glucose profiles to estimate intraday glycemic variability, rather than using 5-minute data points from CGM. Our data (Table 10) showed that CGM-derived metrics of glycemic variability tended to remain associated with diabetes complications after adjusting for HbA_1c_ (and indeed many other potential confounders). However, only one longitudinal study investigated this association, finding CV was associated with baroreflex sensitivity (CAN) after adjusting for HbA_1c_. Therefore, further longitudinal data are required to investigate the glucose variability hypothesis.

### Clinical Implications

Large-scale longitudinal studies have shown that CGM-users have lower HbA_1c_ levels, less hypoglycemia, and more TIR compared to non-CGM cohorts (13, 77). Thus, it would be expected for CGM use to translate longitudinally to reduced complications risk. CGM offers the unique ability to measure glucose levels in situations such as during sleep or exercise. Some systems have no requirement for daily capillary finger-prick tests, which patients appreciate. A survey of 3461 people with T1D or T2D identified TIR as the second most important of all factors that had a “big impact” on daily life with diabetes, while food choices were number one (78). CGM also alleviates the fear of hypoglycemia, which is a major barrier to exercise, dieting, and intensified treatment regimens (79), allowing for early glycemic management. This early management may be crucial in preventing future complications due to the “legacy effect.” A more than 66 000 person-year follow-up of the UKPDS RCT studied the effects of intensive glycemic management on the development of diabetes complications. It demonstrated that early intensive glycemic management resulted in risk reductions that persisted far beyond the transient HbA_1c_ level differences between groups (80). Importantly, the integration of CGM also allows for more sophisticated use of insulin pump therapy, the gold standard for glycemia management.

Health care systems must also adapt to allow for widespread CGM use. This includes education and training around consensus reporting and interpretation of results, their implications on treatment adjustments, as well as follow-up guidelines (81). The ATTD Congress recommends the use of ambulatory glucose profile reports to aid this interpretation (14). Guidelines for when and how often to use CGM also requires further investigation, as Vigersky et al (82) found that periodic 14-day courses of CGM every 3 months would still adequately inform changes and responses to treatment as well as lasting behavioral changes. This may be aided by further cost-effectiveness analysis, which as it stands is extremely variable with different devices, patterns of use, and populations (83, 84).

### Strengths and Limitations

**Figure 4.**
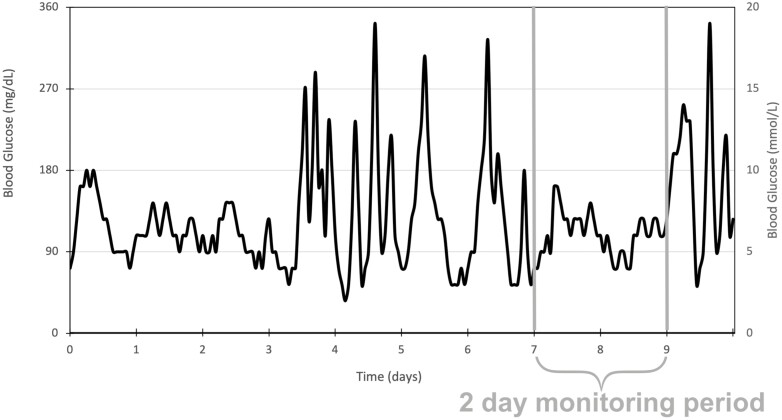
Limitation of short continuous glucose monitoring (CGM) periods. Most studies included in this review have CGM periods of 2 to 3 days. This diagram demonstrates how the data from this period may not be representative of the participants’ overall glycemic management.

**Figure 5.**
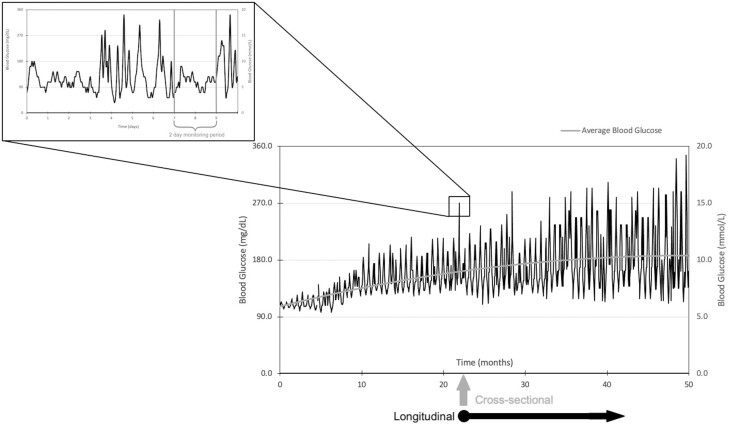
Observational vs longitudinal studies. Thirty out of the 34 papers included in this review used cross-sectional study designs. Diabetes complications are the results of years of altered glycemia. This diagram further illustrates how data from a single point in time (as in a cross-sectional study) may misrepresent the preceding months of data that are causative of the disease outcome. Longitudinal studies may be able to provide a more comprehensive analysis of the associations between different metrics of glycemia and the risk of diabetes complications.

This review had several limitations. First, there was considerable heterogeneity between studies, in the selection of study participants, treatment adjustments, and reporting of data. Thus, a meta-analysis was not possible, making the data more difficult to interpret. Second, 30 out of 34 papers were cross-sectional study designs, meaning that causal relationships between CGM-derived measurements and outcomes cannot be proven. Third, the ATTD Congress suggests longer than 14-day periods of CGM (14). Most studies used only 48 to 72 hours, which may not be representative of long-term control and therefore undermine any associations with long-term complications. This may not be the case in T2D populations, which have less glycemic variability than in T1D, and thus, patterns of glycemia are more reproducible on a day-to-day basis. Shorter monitoring periods in this cohort are therefore more likely to be representative of longer-term patterns of glycemia, which would better correlate with complication development (85). Fourth, some studies included individuals with short diabetes durations, which reduces the likelihood of diabetes-related complications, and therefore underestimates the effect of each marker of glycemic variability on the development of diabetes complications long term. Fifth, there was a distinct lack of data relating to T1D, which is important given the large use of CGM particularly in youth with T1D. While 11 studies involved populations with T1D, this totaled only 663 participants. Sixth, many studies inferred the presence of disease via measuring risk factors or markers of disease rather than definitive outcomes. Finally, while specific populations such as pregnancy were excluded to increase the generalizability and applicability of this review’s findings, this may have led to the exclusion of relevant studies. In particular, publications involving insulin pumps were excluded because their effects on glycemic management may have altered the development of diabetes complications above that of the effect of CGM alone. While this allowed for better isolation of the effects of CGM on diabetes complications, it unrealistically excludes the effect that therapy may have on markers of glycemic variability. Databases such as Web of Science and gray literature were not searched, which may similarly exclude studies. The chief strength of this review includes the number of studies and broad range of complication outcomes included (Figs. 4 and 5).

## Conclusion

Recent technological advancements in CGM present an exciting prospect for the future of cost-effective and equitable diabetes management. This literature review aimed to summarize the existing evidence for the direct link between CGM-derived metrics of glycemic management and complications of diabetes. As per the recommendations from the ATTD Congress, TIR was consolidated as an important metric; however, evidence was weaker for CV. While higher glycemic variability and lower TIR tended to be associated with diabetic complications, future research, particularly in the form of longitudinal studies, meta-analyses, and RCTs, are required to better evaluate relationships between these CGM-derived metrics and all diabetes complications, especially in T1D. Future studies should also consider the effect of closed-loop pump therapy on the development of diabetes complications.

## Data Availability

Some or all data generated or analyzed during this study are included in this published article or in the data repositories listed in “References.”

## Abbreviations

ATTD: Advanced Technologies & Treatments
BGL: blood glucose level
CAN: cardiac autonomic neuropathy
CGM: continuous glucose monitoring
CIMT: carotid intima-media thickness
CONGA: continuous overall net glycemic action
CV: coefficient of variation for glucose
DCCT: Diabetes Control and Complications Trial
EDIC: Epidemiology of Diabetes Interventions and Complications
eGFR: estimated glomerular filtration rate
GMI: glucose management indicator
HbA_1c_: glycated hemoglobin A_1c_
HBGI: high blood glucose index
LADA: latent autoimmune diabetes of adulthood
LBGI: low blood glucose index
MAGE: mean amplitude of glycemic excursions
RCT: randomized controlled trial
SD: standard deviation of blood glucose levels
TAR: time above range
TBR: time below range
TIR: time in range
T1D: type 1 diabetes
T2D: type 2 diabetes
UKPDS: United Kingdom Prospective Diabetes Studies.
